# Supplementary material for: Regulation of Age-Related Lipid Metabolism in Ovarian Cancer
Source: Int J Mol Sci. 2025 Jan 1;26(1):320. doi: 10.3390/ijms26010320 (PMC11720209; doi:10.3390/ijms26010320)
Supplement: Supplementary file 1 [file ijms-26-00320-s001.zip › Table S1 Probes list for RT-qPCR_Huang.pdf]

Supplementary Table S1. TaqMan probes for RT-qPCR

Gene ID	Cat #	Manufacturer
S100a8	Rn00587579_g1	ThermoFisher
S100a9	Rn00585879_m1	ThermoFisher
Il1rl1	Rn01640664_m1	ThermoFisher
Lcn2	Rn00590612_m1	ThermoFisher
C3	Rn00566466_m1	ThermoFisher
Hba-a1	Rn01789798_s1	ThermoFisher
Fcna	Rn00580247_m1	ThermoFisher
Pnpla3	Rn01502361_m1	ThermoFisher
GAPDH	Rn01775763_g1	ThermoFisher
